# History of Pulmonary Tuberculosis Accelerates Early Onset and Severity of COPD: Evidence from a Multicenter Study in Romania

**DOI:** 10.3390/jcm14175980

**Published:** 2025-08-24

**Authors:** Ramona Cioboata, Silviu Gabriel Vlasceanu, Denisa Maria Mitroi, Ovidiu Mircea Zlatian, Mara Amalia Balteanu, Gabriela Marina Andrei, Viorel Biciusca, Mihai Olteanu

**Affiliations:** 1 Department of Pneumology, University of Medicine and Pharmacy, 200349 Craiova, Romania; ramona_cioboata@yahoo.com (R.C.); gabriela.andrei@umfcv.ro (G.M.A.); biciuscaviorel@gmail.com (V.B.); mihai.olteanu@umfcv.ro (M.O.); 2 Department of Pneumology, Victor Babes University Hospital, 200515 Craiova, Romania; 3 Department of Microbiology, “Carol Davila” University of Medicine and Pharmacy, 050474 Bucharest, Romania; silviu.vlasceanu@drd.umfcd.ro; 4 Department of Thoracic Surgery, Marius Nasta Pneumology Institute, 050159 Bucharest, Romania; 5 Doctoral School, University of Medicine and Pharmacy, 200349 Craiova, Romania; denisa_maria2@yahoo.com; 6 Department of Microbiology, University of Medicine and Pharmacy, 200349 Craiova, Romania; 7 Department of Pulmonology, Faculty of Medicine, Titu Maiorescu University, 031593 Bucharest, Romania; 8 Department of Pneumology, Marius Nasta Pneumology Institute, 050159 Bucharest, Romania

**Keywords:** chronic obstructive pulmonary disease, pulmonary tuberculosis, TB, COPD exacerbations, smoking, LMICs, chronic respiratory disease, risk factors

## Abstract

**Background:** Pulmonary tuberculosis (TB) is increasingly recognized as a risk factor for chronic obstructive pulmonary disease (COPD), but its impact on COPD onset and severity remains poorly characterized, particularly in low- and middle-income countries. This multicenter study aimed to assess the impact of prior pulmonary TB on COPD onset, severity, the timing of the first severe exacerbation, and progression among Romanian patients with and without a history of pulmonary TB. **Methods**: This retrospective multicenter study included adults hospitalized for their first severe COPD exacerbation at two tertiary care centers in Romania between April 2020 and April 2025. Patients were grouped based on smoking status and prior TB history. Propensity score matching was used to control for confounding factors. Clinical characteristics, spirometry, and radiological TB patterns were analyzed comparatively between patients with prior TB and TB-naïve patients. **Results**: Among 403 COPD patients, those with prior TB had significantly earlier COPD onset (mean age 48.67 ± 6.42 vs. 65.61 ± 5.14 years in smokers, *p* < 0.001) and shorter intervals to their first severe COPD exacerbation compared to patients without prior TB (6.35 ± 4.71 vs. 15.14 ± 6.93 years in smokers, *p* < 0.001). COPD prevalence was higher among TB survivors compared to those without TB history, especially in smokers (OR = 5.73; 95% CI, 3.30–9.94, *p* < 0.001), versus non-smokers (OR =2.23; 95% CI, 1.37–3.64, *p* = 0.001). Radiological severity of TB lesions significantly influenced COPD prevalence among smokers (OR = 10.79, *p* < 0.001). **Conclusions:** Prior pulmonary TB substantially accelerates COPD onset, exacerbation timing, and disease severity, particularly in smokers. This multicenter comparative study demonstrates that prior pulmonary TB significantly accelerates COPD onset, exacerbation timing, and disease severity, especially among smokers. Recognizing TB history as a significant COPD risk factor underscores the importance of targeted COPD screening and tailored management in populations with high TB prevalence.

## 1. Introduction

Chronic obstructive pulmonary disease (COPD) ranks among the leading causes of mortality globally, disproportionately affecting low- and middle-income countries (LMICs) [1]. Its prevalence continues to rise due to increased exposure to key risk factors such as smoking and biomass smoke, along with demographic shifts towards older populations [2]. These factors may also lead to a higher incidence of COPD among non-smokers, influencing both preventive measures and therapeutic approaches [3]. Currently, COPD has an estimated global prevalence rate of 10.3%, with a 95% confidence interval (CI) ranging from 8.2% to 12.8% [1,4].

Notably, pulmonary tuberculosis (TB), itself highly prevalent in LMICs, has been recognized as an important risk factor for COPD development, exacerbation, and accelerated progression.

In 2023, an estimated 8.2 million people worldwide received a new TB diagnosis, a notable increase from 7.5 million in 2022 and 7.1 million in 2019, and significantly higher compared to the 5.8 million and 6.4 million cases reported in 2020 and 2021, respectively. This recent surge likely reflects a backlog of undiagnosed TB cases from earlier years, driven by delays in diagnosis and treatment resulting from COVID-19 disruptions [5,6].

According to the WHO Global Tuberculosis Report 2024, Romania not only has the highest TB incidence in the European Union (EU) but also faces challenges with TB control compared to neighboring countries. In recent years, Romania has contributed to over 20% of the total TB burden in the EU, despite having only about 4% of the EU’s population [5,7].

TB incidence in Romania remains approximately 4–5 times higher than the EU average, with persistent challenges in TB control, including higher rates of drug-resistant TB [8]. COPD prevalence in Romania is also among the highest in Central and Eastern Europe, with national surveys indicating that over 8% of adults are affected, and underdiagnosis remains a significant issue.

Romania faces a dual public health challenge: it has the highest TB incidence in the EU and also reports a high prevalence of COPD. COPD affects over 8% of Romanian adults, with significant underdiagnosis, and the country has among the highest COPD mortality rates in Europe [9,10,11].

COPD and TB share a complex, bidirectional relationship. Individuals with a history of TB have a 1.7- to 4-fold increased risk of developing COPD, independently of smoking and other common risk factors [12,13,14], and display significantly reduced lung function parameters, such as forced expiratory volume (FEV_1_) and forced vital capacity (FVC), compared to those without prior TB [4,8,9]. This association is particularly significant in low- and middle-income regions, where TB is prevalent and contributes substantially to the COPD burden [13,15,16]. COPD patients with a history of TB are diagnosed at a younger age, experience more frequent hospitalizations, and have worse respiratory outcomes, including reduced FEV_1_ and higher arterial CO_2_, which accelerate disease progression and increase mortality. TB-related lung damage and chronic inflammation worsen these outcomes, while shared risk factors like smoking and socioeconomic hardship further complicate management. Notably, COPD patients are also at increased risk of developing active TB, underscoring the need for integrated prevention and regular monitoring in this population [17,18]. Mechanistically, post-tuberculosis lung disease (PTLD) can result in chronic structural and immunological alterations that accelerate COPD progression. TB-related lung damage often includes fibrosis, bronchiectasis, and parenchymal destruction, which permanently reduce lung function and predispose the body to airflow limitation. Additionally, chronic inflammation persisting after TB resolution leads to ongoing remodeling of the airways and alveolar spaces. Impaired mucociliary clearance, persistent immune dysregulation, and increased susceptibility to secondary infections further compound the risk of early and severe COPD in TB survivors [19,20,21].

COPD and TB individually represent substantial global health and economic burdens, particularly in low- and middle-income countries where healthcare resources are limited and disease prevalence remains high [13].

The coexistence of COPD and TB poses significant diagnostic and therapeutic challenges due to overlapping clinical features, shared risk factors, and compounded lung damage [13,15,22]. These challenges are particularly pronounced in Romania, which not only bears the highest incidence of TB in the European Union but is also experiencing a rising burden of COPD [5,7]. Resource limitations, elevated comorbidity rates, and restricted access to integrated care exacerbate the strain on the Romanian healthcare system [23,24,25]. The relevance of investigating a multicenter Romanian population extends beyond the national context, as Romania exemplifies regions with both high TB and COPD prevalence, constrained healthcare resources, and dynamic epidemiological transitions. By selecting the largest pneumology hospitals in southern Romania, Victor Babes University Hospital in Craiova and the Marius Nasta Institute of Pneumology in Bucharest, our study captures a representative patient population from high-burden, resource-limited settings. Despite the significant clinical and public health impact of these comorbidities, there is a critical lack of country-specific data on how prior TB influences COPD outcomes in Romania. This study aims to address this gap by providing robust evidence on the impact of prior TB on COPD severity, progression, and prognosis, thereby informing tailored preventive and management strategies for similar high-burden settings.

## 2. Materials and Methods

### 2.1. Study Design and Setting

This retrospective study was conducted in two tertiary-care centers in Romania: Victor Babes University Hospital, Craiova, and the Marius Nasta Institute of Pneumology, Bucharest, from April 2020 to April 2025. The study population comprised adults aged 40 years and older who were consecutively hospitalized for their first severe COPD exacerbation in the Pneumology Departments of these institutions. For each patient, only the initial (index) admission during the study period was included in the analysis.

To provide a comparative baseline and to evaluate the specific impact of prior pulmonary tuberculosis on clinical outcomes, a control group was established, consisting of adults hospitalized during the same period without a COPD diagnosis, as confirmed by normal spirometry results and clinical assessment. All procedures adhered to the Declaration of Helsinki and received approval from the Ethics Committees of both institutions (Marius Nasta Institute of Pneumology: No. 2721/10.02.2025; Victor Babes University Hospital: No. 5628/16.04.2025). Written informed consent for the anonymized use of clinical data for research purposes was obtained from each patient upon admission to these academic institutions.

### 2.2. COPD Diagnosis and Severity Classification

COPD was diagnosed by pulmonologists on the basis of chronic respiratory symptoms and relevant exposures, and confirmed by spirometry in accordance with Global Iitiative for Chronic Obstructive Lung Disease (GOLD) criteria [1]. To maintain consistency in classification, spirometry assessments conducted after the exacerbation, before patient discharge from the hospital, were utilized. A post-bronchodilator FEV_1_/FVC ratio < 0.70 was mandatory for inclusion. Disease severity was staged per GOLD using percent-predicted FEV_1_: stage I (≥80%), stage II (50–79%), stage III (30–49%), and stage IV (<30%).

### 2.3. Tuberculosis Diagnosis and Treatment History

A history of pulmonary tuberculosis was determined for each patient using records from the national TB database. The diagnosis required clinical symptoms consistent with active pulmonary TB, radiographic findings indicative of active disease (infiltrates, nodular lesions, cavities), and microbiological confirmation with at least one sputum sample positive for acid-fast bacilli (AFB) by Ziehl–Neelsen staining and subsequent culture confirmation of *Mycobacterium tuberculosis* on Lowenstein–Jensen medium according to national guidelines. A new TB case was defined as a patient who had either never received anti-tuberculosis therapy or had received treatment for less than one month. A relapse case was defined as a patient previously treated for TB, declared cured or who had completed therapy, but subsequently diagnosed again with bacteriologically confirmed active TB. Only patients meeting all diagnostic criteria (clinical, radiographic, microbiologic) were classified as having a history of pulmonary TB.

All TB cases included in this study were drug-sensitive and had completed the standard six-month treatment regimen recommended by the Romanian National Tuberculosis Control Program. The regimen comprised a two-month intensive phase of daily isoniazid, rifampicin, pyrazinamide, and ethambutol, followed by a four-month continuation phase with isoniazid and rifampicin. Documentation detailing the treatment regimen, classification as a new case or relapse, radiological form of TB, and adherence data were recorded in the national TB database. Follow-up evaluations were conducted in outpatient settings in line with national protocols, ensuring consistency and high standards in patient care and data collection.

### 2.4. Inclusion and Exclusion Criteria

The main objective of this study was to determine whether a history of pulmonary TB influences the onset of COPD and the severity and timing of the first severe exacerbation in affected patients. To achieve this, we specifically examined the relationship between prior TB, age at COPD diagnosis, time to first severe COPD exacerbation, and clinical severity. Patients were stratified into four groups according to their smoking status (ever-smokers vs. never-smokers) and TB history (prior TB vs. no TB) to distinguish the independent and combined effects of TB and smoking. The inclusion of a control group without COPD, also stratified by TB history, provided a relevant baseline to evaluate the unique contribution of previous TB to the risk and characteristics of COPD. This design allowed for a clear analysis of how TB history, in combination with smoking and radiological TB patterns, may accelerate COPD onset and increase the risk of earlier and more severe exacerbations in the Romanian population.

Initially, 4273 hospitalized patients with severe COPD exacerbations were screened. To minimize selection bias, consecutive patients admitted to two major tertiary pneumology centers in Romania were included. Strict inclusion criteria were applied consistently across centers, and standardized protocols for COPD diagnosis and severity assessment were followed, including spirometric confirmation. After applying exclusion criteria prior history or hospitalization for severe COPD exacerbation, incomplete medical records, HIV-positive status, pulmonary malignancy, unconfirmed or uncertain history of pulmonary TB, active pulmonary TB at admission, and abandonment of TB treatment, a total of 403 patients experiencing their first severe COPD exacerbation were included in the final analysis. Severe exacerbations were defined as episodes of worsening respiratory symptoms necessitating hospitalization.

Controls were selected from the same institutions during the same time frame to ensure comparability. The control group comprised adults aged 18 years or older who attended Victor Babes University Hospital (Craiova) or the Marius Nasta Institute of Pneumology (Bucharest) during the study period (April 2020 to April 2025) for pre-employment assessments or systematic TB contact screening, as mandated by the National Tuberculosis Program.

A TB contact case refers to an individual who has had exposure to a person with active tuberculosis, such as a household member or workplace colleague, and is screened according to national TB control protocols.

**Figure 1:**
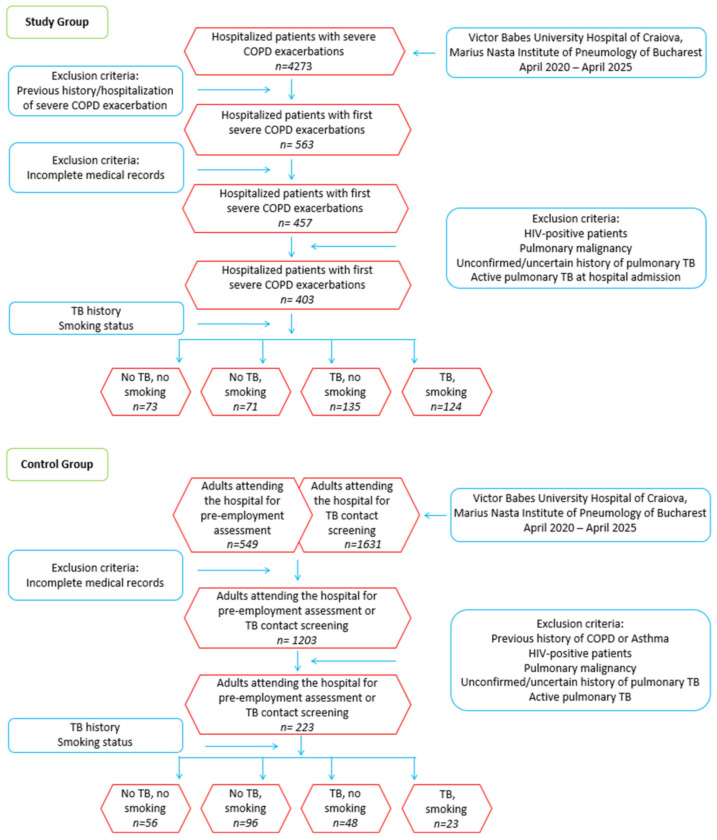
Flowchart of study participant selection and group stratification. COPD: chronic obstructive pulmonary disease; TB: tuberculosis.

Controls were included regardless of TB history and were stratified into two subgroups: those with prior pulmonary TB and those without, facilitating the assessment of the independent impact of previous TB on lung function. All control subjects had no clinical or spirometric evidence of COPD, confirmed by a normal FEV_1_/FVC ratio and absence of respiratory symptoms. Applying these rigorous selection criteria, the final control group consisted of 223 participants, including 71 with prior TB and 152 without any TB history (Figure 1). This structured approach allowed for robust and direct comparisons of baseline characteristics, pulmonary function, and clinical outcomes independently of COPD status, thereby strengthening the validity and reliability of the analysis.

From the electronic patient records, we collected demographic data (age, gender) and clinical history, including age at COPD diagnosis, age at first severe exacerbation, details of COPD treatment, COPD stage according to GOLD criteria, and spirometric parameters. Additionally, we recorded age at TB diagnosis, type of TB case (new case or relapse), radiological form of TB, smoking history, and body mass index. In addition, we documented comorbidities such as obesity, cardiovascular disease and diabetes. Smoking exposure was quantified using the pack-year metric calculated as (cigarettes per day ÷ 20) × years smoked. Laboratory results, such as complete blood count and inflammatory markers, were also extracted.

Patients who were lost to follow-up or had incomplete data were excluded from the final analysis to maintain the integrity and reliability of our findings.

### 2.5. Statistical Analysis

Our study was adequately powered (≥80%) to detect moderate effect sizes for continuous variables and odds ratios ≥ 2.0 for binary outcomes, given our final sample size of 403 patients.

Continuous variables were presented as mean ± standard deviation. Categorical variables were presented as percentages. Comparison of groups was performed either by Student’s *t*-test for continuous variables (after checking the normality of distribution) or by Chi squared test with Fisher’s correction (for categorical variables), e.g., when comparing COPD patients from the four subgroups defined by TB history and smoking status or when comparing COPD patients with controls.

While we analyzed key subgroups stratified by smoking status and prior TB history, further subgroup analyses were not performed due to sample-size considerations and to avoid compromising statistical power.

Effect sizes are reported as odds ratios (ORs) with corresponding 95% confidence intervals (CIs) for binary outcomes and as mean differences with standard deviations (SDs) for continuous variables.

In observational studies, where prior TB exposure is not randomly assigned, systematic differences often exist between those with and without a history of TB. Patients who developed TB in the past may differ from TB-free individuals in important baseline characteristics such as age, sex, body mass index (BMI), cardiovascular comorbidities, and socioeconomic factors that themselves influence COPD onset, severity, or progression. These baseline differences represent potential confounding variables that can bias the estimated effect of TB on COPD outcomes.

Propensity score matching (PSM) addresses this challenge by creating analytic groups of TB-exposed and unexposed individuals with similar distributions of observed baseline characteristics [26]. The propensity score, defined as the probability of having a history of TB given the observed covariates, allows for the formation of matched sets where differences in baseline risk factors are minimized. This mimics some features of randomized controlled trials, thus enabling a more accurate estimation of the independent effect of TB history on COPD risk and severity. Without such matching, any observed associations between prior TB and COPD outcomes could be confounded by these imbalances, leading to misleading or exaggerated results [27].

Therefore, we used propensity score matching for matching cases with COPD with controls respective to exposure to tuberculosis.

The propensity score (the probability of having a history of tuberculosis) was estimated using a probit regression model incorporating standardized age, gender, BMI, atrial fibrillation, and heart failure as covariates. We selected a probit regression model to estimate the propensity score, as this is the default algorithm in Stata 17.0 SE statistical software (StataCorp Ltd., College Station, TX, USA) psmatch2 command for treatment-effect matching [28]. Matching was performed using radius matching with a caliper of 0.1 on the propensity score as previously suggested [29], as implemented in the psmatch2 module of Stata [28]. Matching was performed with replacements and limited to the region of common support. All 183 patients with a history of TB (treated) and 129 controls (untreated) remained in the matched sample, with no observations dropped due to lack of support. Covariate balance before and after matching was evaluated using standardized mean differences (SMDs), *t*-tests, and variance ratios for each covariate, as well as summary measures (pseudo-R^2^, mean and median SMD, Rubin’s B and R). Balance was considered adequate if SMDs were <10%, Rubin’s B < 25%, and Rubin’s R between 0.5 and 2.

To quantify the independent association between prior pulmonary tuberculosis and COPD outcomes, we conducted multivariate logistic regression analyses within propensity-score matched cohorts. COPD prevalence (binary outcome) was modeled as a function of TB history, adjusting for residual covariate imbalances and clustered match-pair structure. The logistic model estimated adjusted odds ratios (ORs) with 95% confidence intervals. We ensured adherence to recommended practices by including only key predictors (e.g., age, sex, BMI, smoking status, cardiovascular comorbidities).

## 3. Results

A total of 403 consecutive patients hospitalized for their first severe COPD exacerbation were analyzed and stratified into four exposure groups based on smoking status and prior TB history: no-TB/no-smoking (*n* = 73), TB/no-smoking (*n* = 71), no-TB/smoking (*n* = 135), and TB/smoking (*n* = 124). Demographic and clinical characteristics across these groups are summarized in Table 1. We compared TB and No TB patients by Student’s t-test or Chi squared test in the smoking and non-smoking strata.

Among non-smokers, patients with prior TB history were significantly younger (53.57 ± 12.46 vs. 62.15 ± 16.87 years; *p* < 0.001), diagnosed with COPD at a younger age (48.67 ± 6.42 vs. 53.14 ± 13.03 years; *p* < 0.001), and experienced their first severe COPD exacerbation significantly sooner after diagnosis (9.36 ± 3.97 vs. 17.43 ± 12.48 years; *p* < 0.001) compared to non-smokers without prior TB. However, COPD severity staging (GOLD stages 2–3), BMI, obesity prevalence, and comorbidities (high blood pressure, heart failure, atrial fibrillation, diabetes mellitus) were comparable between TB-exposed and unexposed non-smokers.

Among smokers, patients with prior TB history similarly demonstrated significantly younger mean age (54.40 ± 8.46 vs. 57.74 ± 18.47 years; *p* = 0.045), earlier initiation of smoking (20.52 ± 4.43 vs. 24.05 ± 8.94 years; *p* < 0.001), longer cumulative smoking duration (38.89 ± 11.96 vs. 33.71 ± 16.77 years; *p* = 0.002), intensity of smoking (33.14 vs. 18.69 pack/years; *p* = 0.029), and notably younger age at COPD diagnosis (47.43 ± 5.12 vs. 65.61 ± 5.14 years; *p* < 0.001). Furthermore, smokers with prior TB had a significantly shorter interval between COPD diagnosis and their first severe exacerbation (6.35 ± 4.71 vs. 15.14 ± 6.93 years; *p* < 0.001). COPD staging, BMI, obesity, and comorbidities were generally balanced between groups, although diabetes mellitus prevalence was significantly lower in smokers with prior TB (19.73% vs. 29.34%; *p* = 0.049).

Pharmacological therapy utilization varied significantly, particularly among smokers: those with prior TB were significantly more likely to receive bronchodilator therapy (LABA: 21.37% vs. 1.48%; *p* = 0.004) and inhaled corticosteroids plus bronchodilator combinations (ICS + LABA: 49.66% vs. 22.16%; *p* < 0.001). Additionally, non-smokers with prior TB were significantly more likely to receive ICS + LABA + LAMA therapy compared to non-smokers without prior TB (29.51% vs. 10.08%; *p* < 0.001).

Pulmonary function parameters were significantly influenced by a history of TB and smoking status. Among non-smokers, subjects with prior pulmonary TB had significantly lower percent predicted FEV1 (60.79% ± 25.00% vs. 68.24% ± 26.49%; *p* = 0.012) compared to those without TB. Percent predicted FVC was also significantly lower in the TB group (72.66% ± 19.31% vs. 80.05% ± 19.66%; *p* = 0.001). However, the FEV1/FVC ratio was not significantly different between groups without smoking history (*p* = 0.139).

Among smokers, prior TB infection was associated with marked reductions in pulmonary function. Smokers with prior TB had significantly lower FEV1% (55.10% ± 19.44% vs. 76.36% ± 26.88%; *p* < 0.001) and significantly reduced FVC% (70.09% ± 18.29% vs. 84.46% ± 22.95%; *p* < 0.001). The FEV1/FVC ratio was also significantly lower in smokers with prior TB compared to smokers without prior TB (*p* < 0.001). These findings indicate that prior pulmonary TB significantly exacerbates smoking-related impairment in pulmonary function.

The comparison between COPD cases and controls (Table 2) revealed significant differences in several demographic and clinical characteristics. Patients with COPD were significantly older than controls (mean age 59.72 ± 11.68 vs. 48.16 ± 17.86 years, *p* < 0.001) and exhibited a higher prevalence of previous tuberculosis (64.27% vs. 31.84%, OR = 3.85, *p* < 0.001). Although overall smoking prevalence did not differ significantly between groups, COPD patients had a significantly longer smoking duration (40.38 ± 11.55 vs. 29.18 ± 17.10 years, *p* < 0.001) and began smoking at an older age (24.23 ± 8.65 vs. 19.33 ± 2.58 years, *p* < 0.001). There were no significant differences in gender distribution, BMI, obesity rates, or the prevalence of comorbidities such as high blood pressure, heart failure, atrial fibrillation, or diabetes mellitus.

Collectively, these results demonstrate that prior pulmonary TB significantly influences the age at COPD onset and accelerates progression toward severe exacerbation, with amplified effects in smokers, highlighting the clinical importance of recognizing TB history as a major determinant of COPD severity and progression.

### 3.1. Propensity Score Distribution and Overlap

To minimize potential confounding from baseline differences between patients with and without a history of TB, PSM was employed using demographic and clinical covariates (age, sex, body mass index, atrial fibrillation, heart failure). The propensity score defined as the probability of having a history of TB given observed covariates was estimated using probit regression. Matching of TB-exposed (“treated”) and unexposed (“untreated”) participants was conducted via radius matching with a caliper of 0.1, implemented in Stata’s psmatch2 module [28,29]. Matching was performed with replacement and restricted to the region of common support.

**Figure 2:**
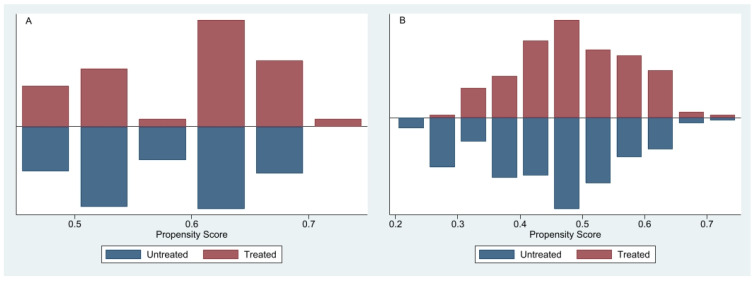
Distribution of propensity scores for treated and untreated participants in (**A**) never-smokers and (**B**) ever-smokers. Stacked histograms compare the density of individuals with and without a history of tuberculosis (treated vs. untreated) across strata of the estimated propensity score, before matching. The degree of overlap illustrates the extent of common support between groups and the potential for effective matching.

The distributions of propensity scores before matching, presented separately for never-smokers (Figure 2A) and ever-smokers (Figure 2B), indicate varying degrees of overlap. In the never-smoker cohort, treated participants had higher propensity scores, whereas untreated participants clustered toward lower and intermediate scores, resulting in moderate overlap sufficient for reliable treatment-effect estimation. In contrast, the ever-smoker cohort exhibited substantial overlap, particularly within propensity score ranges of 0.4–0.6, facilitating effective matching and robust estimation of TB’s independent impact on COPD outcomes [26].

Covariate balance after matching was evaluated using standardized mean differences (SMDs), variance ratios, and Rubin’s B and R statistics. Balance was considered adequate if SMDs were <10%, Rubin’s B < 25%, and Rubin’s R between 0.5 and 2, ensuring comparability across matched groups [30].

After matching, among never-smokers (*n* = 312), COPD prevalence was significantly higher in those with prior TB (73.8%) compared to matched controls without TB (55.7%), resulting in an average treatment effect on the treated (ATT) of 18 percentage points (SE = 5.6, *p* = 0.001) for those exposed to TB. This corresponded to an odds ratio (OR) of 2.23 (95% CI: 1.37–3.64), indicating a more than twofold higher risk of COPD associated with previous TB infection in the absence of smoking (Table 3).

Among ever-smokers (*n* = 314), the association was even stronger. COPD prevalence was 84.4% among patients with prior TB compared to 48.5% in matched smokers without prior TB, translating to an ATT of 36 percentage points (SE = 5.1, *p* < 0.001). This yielded an OR of 5.73 (95% CI: 3.30–9.94), underscoring that previous TB substantially amplifies the risk of developing COPD, particularly when coupled with smoking exposure. Collectively, these matched analyses highlight prior pulmonary TB as a robust and independent risk factor for COPD, significantly elevating disease risk and severity across smoking strata.

### 3.2. Radiological TB Patterns and Spirometric Outcomes

We analyzed radiological characteristics of patients with a confirmed history of pulmonary TB, stratified according to lesion distribution (unilateral vs. bilateral) and lesion type (cavitary vs. non-cavitary). These radiological patterns were then correlated with COPD prevalence and the time elapsed until the first severe COPD exacerbation, separately evaluated for smokers (*n* = 190) and non-smokers (*n* = 183), representing the complete population of COPD patients with a documented prior TB history included in this analysis.

Our findings highlight that specific radiological characteristics of prior pulmonary TB particularly lesion distribution (unilateral vs. bilateral) and lesion type (cavitary vs. non-cavitary) significantly influence COPD prevalence, with effects strongly dependent on smoking status.

Among non-smokers, the COPD prevalence was similarly high irrespective of lesion distribution (unilateral: 70.69%; bilateral: 75.20%; OR: 1.26, *p* = 0.519) or cavitation presence (non-cavitary: 73.97%; cavitary: 72.98%; OR: 0.95, *p* = 0.902), suggesting minimal additional impact of radiological severity when smoking is absent (Table 4).

In contrast, among smokers, bilateral TB significantly increased COPD prevalence (87.38%) compared to unilateral involvement (39.08%; OR: 10.79, *p* < 0.001). Similarly, cavitary TB markedly elevated COPD prevalence among smokers (90.00%) relative to non-cavitary forms (56.43%; OR: 6.95, *p* < 0.001). These differences indicate a pronounced synergy between smoking and severe TB-related lung injury, emphasizing the compounded risk posed by smoking alongside extensive or cavitary TB lesions.

The time until the first severe COPD exacerbation was not significantly affected by TB lesion characteristics in either smokers or non-smokers (all *p* > 0.05), suggesting that radiological severity influences COPD prevalence more clearly than it impacts the timing of disease exacerbations.

## 4. Discussion

This multicenter retrospective study provides essential insights into the impact of previous pulmonary TB on the clinical outcomes and severity of COPD among Romanian patients. Consistent with prior studies, our findings underscore that a history of pulmonary TB significantly accelerates COPD onset and severity, independently of other risk factors such as smoking [12,13,14]. Notably, patients with prior TB experienced their first severe COPD exacerbation significantly earlier (mean 6.35 ± 4.71 years vs. 15.14 ± 6.93 years, *p* < 0.001) and displayed markedly worse pulmonary function, with significantly lower FEV_1_ (55.10% ± 19.44 vs. 76.36% ± 26.88, *p* < 0.001) compared to patients without TB.

Our findings are in strong concordance with recent international studies, further validating the detrimental effect of prior TB on lung function and COPD outcomes. A prospective cohort study demonstrated that individuals with a history of TB exhibited significantly reduced FEV_1_% and FEV_1_/FVC ratios, as well as more severe airflow limitation, compared to controls without prior TB [31]. This study found a clear dose–response relationship between declining lung function and the probability of developing COPD among those with previous TB. Consistent with their observations, our cohort revealed significantly lower FVC (70.09% ± 18.29 vs. 84.46% ± 22.95, *p* < 0.001) and FEV1 (60.79% ± 25.00 vs. 68.24% ± 26.49; *p* = 0.012), and younger age at COPD diagnosis (47.43 ± 5.12 vs. 65.61 ± 5.14 years, *p* < 0.001) in TB-exposed patients, supporting the concept that TB accelerates both the onset and severity of COPD. Furthermore, our findings corroborate the association between a history of TB and not only physiological impairment but also an elevated risk of acute exacerbations and more severe clinical symptoms in patients with COPD.

Similarly, a large population-based matched cohort study showed that TB survivors had markedly higher COPD incidence rates (36.7/1000 vs. 18.8/1000 person-years, *p* < 0.001) and were at significantly increased risk for COPD-related hospitalizations compared to matched controls (aHR for hospitalization, 2.03; 95% CI, 1.81–2.27) [32]. Our study corroborates these observations by demonstrating a significantly earlier onset of severe COPD exacerbation and a more rapid decline in spirometric parameters among post-TB patients. The increased burden of COPD-related hospitalizations in TB survivors aligns with our finding that post-TB COPD patients have a more severe disease course and worse lung function than their non-TB counterparts [13,33,34].

The accelerated progression and increased severity of COPD observed in post-tuberculosis patients arise from a distinct combination of pathophysiological mechanisms compared to smoking-related COPD. Persistent inflammation and immune dysregulation characterized by elevated cytokines such as IL-1, IL-6, and TNF-α, and heightened immune cell activity remain even after successful TB treatment [17,19,35]. Additionally, increased matrix metalloproteinase activity and neutrophil-driven inflammation contribute to alveolar destruction and fibrotic scarring, promoting airway remodeling and impaired lung function. Structural lung changes including cavitation, fibrosis, bronchiectasis, and parenchymal destruction further exacerbate airflow limitation [36]. Notably, even in patients without visible radiological sequelae, an increased risk of airflow obstruction persists, highlighting subtle underlying pathological changes. Moreover, metabolic alterations such as disrupted fatty acid and tryptophan metabolism and heightened systemic inflammation (elevated IL-6, C-reactive protein) distinguish post-TB COPD from tobacco-related COPD, underscoring the unique nature of this disease subset. Given these distinct mechanisms, there is an evident need for targeted screening and early intervention strategies, especially in TB-endemic regions [37]. Routine spirometric evaluations among TB survivors, irrespective of smoking status, integrated into existing TB control programs, could enable early COPD detection and management [38]. Such an approach may facilitate timely interventions including personalized pharmacotherapy, pulmonary rehabilitation, smoking cessation programs, and vaccination efforts, ultimately mitigating disease progression and improving patient outcomes.

Our study, stratifying COPD risk by smoking status, confirms and expands upon existing evidence identifying prior pulmonary TB as a significant independent risk factor for COPD. We observed a nearly sixfold increase (OR: 5.727, 95% CI: 3.301–9.937, *p* < 0.001) in COPD risk among smokers with a history of TB and more than a twofold increase (OR: 2.233, 95% CI: 1.371–3.640, *p* = 0.001) among non-smokers, underscoring the additive and independent effects of these exposures. These findings align with previous large-scale prospective cohort studies, which similarly reported increased COPD risks in individuals with prior TB, particularly pronounced among smokers. This consistency highlights the global significance of TB as a COPD risk factor across diverse populations [15,32,38,39].

Our results closely parallel findings from systematic reviews and meta-analyses demonstrating that prior TB is associated with a substantially increased risk of developing COPD (OR: 2.59, 95% CI: 2.12–3.15). Notably, these analyses have shown persistent associations even among never-smokers (OR: 2.41, 95% CI: 1.74–3.32), reinforcing our observation that TB independently heightens COPD risk irrespective of smoking status. Collectively, these findings suggest that TB-induced chronic inflammation and structural lung damage significantly contribute to airflow obstruction [38].

Our data agree with previous cross-sectional studies indicating that prior TB independently increases the risk of airflow obstruction (OR: 1.37, 95% CI: 1.13–1.67) after adjusting for smoking and other environmental exposures. The finding that smoking does not substantially modify this association is consistent with our results, further emphasizing TB as an important standalone risk factor for COPD development [40].

Our findings underscore the importance for clinicians and policymakers to recognize prior TB as a critical risk factor for COPD, advocating for routine spirometric assessment and targeted interventions among TB survivors, especially in high-prevalence regions. Propensity score matching effectively controlled for confounders such as age, gender, BMI, and cardiometabolic comorbidities, confirming that previous TB significantly increases COPD prevalence.

Additionally, COPD prevalence varied based on TB radiological characteristics, with smokers having bilateral TB experiencing a notably higher COPD prevalence (87.38%) compared to unilateral forms (39.08%, *p* < 0.001). Similarly, smokers with cavitary TB demonstrated significantly higher COPD prevalence (90.00%) than those with non-cavitary TB (56.43%, *p* < 0.001), emphasizing the critical role of radiological degree of TB severity in COPD progression.

These findings align with recent research highlighting significant radiological variations in pulmonary TB patients with comorbidities, including COPD [20]. Specifically, prior studies noted that COPD patients commonly exhibit radiological findings such as infiltrates and consolidations, potentially contributing to accelerated disease onset and progression [41]. Moreover, ongoing multicenter prospective studies suggest distinct clinical trajectories and outcomes for tuberculosis-associated COPD, reinforcing our observation of radiological severity as an essential predictor of COPD risk and severity [42,43]. Collectively, our findings underline the importance of recognizing specific radiological patterns of prior TB as significant indicators in COPD management, advocating for targeted interventions and regular monitoring of individuals with a history of severe TB forms.

Our study addresses an essential research data gap in Romania, a country with high TB prevalence but limited data on COPD outcomes post-TB infection. These results provide important evidence supporting targeted public health strategies and tailored clinical management for reducing COPD burden among TB survivors.

## 5. Conclusions

This multicenter, propensity score-matched retrospective study demonstrates that a history of pulmonary TB is a significant and independent risk factor for earlier onset, increased severity, and accelerated progression of COPD among Romanian patients. Our findings reveal that individuals with prior TB develop COPD at a younger age, experience their first severe exacerbation sooner, and have more pronounced impairment in pulmonary function particularly among smokers. The radiological severity of previous TB, including bilateral involvement and cavitary lesions, further amplifies the risk and prevalence of COPD, especially when combined with smoking exposure.

Our findings highlight TB history as a critical determinant of COPD risk, emphasizing the need for integrated screening and early management strategies. Clinicians should prioritize spirometric evaluation among TB survivors, even without traditional COPD risk factors. Policymakers are encouraged to integrate post-TB COPD care into national TB programs, focusing on preventive measures, rehabilitation, and vaccination. Future research should include larger, prospective, international cohorts, incorporating pharmacy-refill adherence data and longitudinal spirometry. Additionally, interventional studies evaluating rehabilitation and preventive strategies specifically for post-TB populations are recommended.

## 6. Limitations

This study has several limitations primarily related to its retrospective design. The potential for information bias, including recall or misclassification bias, cannot be fully excluded, as smoking history, symptom onset, and treatment adherence relied largely on patient reports and medical records. Objective adherence measures, such as pharmacy refill data or electronic medication monitoring, were unavailable, and incomplete linkage with national TB registries might have resulted in some missed or misclassified TB histories. Additionally, key confounding factors like vaccination status, biomass exposure, occupational hazards, and socioeconomic variables were not thoroughly assessed, potentially influencing COPD outcomes independently of TB history.

Radiological assessments were conducted retrospectively without blinding evaluators to patients’ TB status, possibly introducing observer bias. Finally, because we included only patients hospitalized for severe COPD exacerbations at specialized tertiary pneumology centers, selection bias might limit the generalizability of our findings. Consequently, associations reported here might differ in magnitude when compared to broader COPD populations managed in primary care or community settings.

Future prospective studies should incorporate diverse clinical environments, objective adherence assessments, comprehensive linkage to national TB databases, and blinded radiological evaluations to address these limitations and enhance the robustness and applicability of our results.

## 7. Future Directions

A nation-wide, prospective, multi-center cohort that captures both hospitalized and community-managed incident COPD cases would markedly improve external validity, enable time-updated exposure assessment including objective pharmacy-refill metrics for treatment adherence and facilitate linkage with national TB surveillance databases. Complementing such a registry, longitudinal lung-function evaluation through repeated spirometry and diffusing-capacity measurements in TB-exposed and TB-naïve patients could reveal divergent trajectories of FEV_1_ decline and gas-exchange impairment, thereby refining risk stratification and guiding personalized follow-up schedules. To translate these insights into practice, pragmatic interventional trials assessing integrated post-TB care bundles combining early pulmonary rehabilitation, optimized inhaled therapy, vaccination, and robust smoking-cessation support are needed to determine whether tailored management can lower exacerbation frequency and enhance quality of life within this high-risk subgroup.

Given the significant global burden and variability of TB and COPD prevalence, future research would benefit substantially from international collaboration to validate our findings across diverse TB/COPD populations. Multinational studies involving countries with varying healthcare infrastructures, TB incidence rates, and COPD management practices would provide broader insight into the relationship between prior pulmonary TB and COPD outcomes. Such collaborative efforts could facilitate the development of universally applicable screening guidelines, preventive measures, and tailored therapeutic strategies, ultimately contributing to global improvements in COPD management among TB survivors.

## Figures and Tables

**Table 1:** Demographic, clinical and paraclinical characteristics of COPD patients stratified by smoking status and history of pulmonary TB.

Variable	No TB, No Smoking *n* = 73	TB, No Smoking *n* = 71	*p* Value	No TB, Smoking *n* = 135	TB, Smoking *n* = 124	*p* Value
Male gender	53.49%	66.12%	0.089	56.89%	61.22%	0.600
Age (years)	62.15 ± 16.87	53.57 ± 12.46	<0.001	57.74 ± 18.47	54.40 ± 8.46	0.045
Smoking time (years)	-	-	-	33.71 ± 16.77	38.89 ± 11.96	0.002
Age at 1st smoke (years)	-	-	-	24.05 ± 8.94	20.52 ± 4.43	<0.001
Packs/year	-	-	-	33.14 ±13.56	18.69 ±13.33	0.029
Age at COPD diagnosis (years)	53.14 ± 13.03	48.67 ± 6.42	<0.001	65.61 ± 5.14	47.43 ± 5.12	<0.001
Time to 1st severe COPD exacerbation (years)	17.43 ± 12.48	9.36 ± 3.97	<0.001	15.14 ± 6.93	6.35 ± 4.71	<0.001
COPD staging	2—42.47% 3—57.53%	2—45.19% 3—54.81%	0.742	2—45.07% 3—54.93%	2—45.17% 3—54.83%	0.9871
FEV_1_ (L)	2.37 ± 0.86	2.17 ± 0.79	0.036	2.59 ± 0.89	1.96 ± 0.74	<0.001
FEV_1_ (%)	68.24 ± 26.49	60.79 ± 25.00	0.012	76.36 ± 26.88	55.10 ± 19.44	<0.001
FVC (L)	3.58 ± 0.76	3.43 ± 0.80	0.096	3.70 ± 0.76	3.31 ± 0.78	<0.001
FVC (%)	80.05 ± 19.66	72.66 ± 19.31	0.001	84.46 ±22.95	70.09 ±18.29	<0.001
FEV_1_/FVC	0.64 ± 0.14	0.62 ± 0.13	0.139	0.69 ± 0.15	0.58 ± 0.13	<0.001
BMI	27.38 ± 4.02	27.86 ± 4.34	0.329	27.36 ± 4.49	28.17 ± 4.33	0.106
Obesity	70.54%	69.40%	0.828	63.47%	72.11%	0.103
HBP	10.08%	12.57%	0.498	10.18%	15.65%	0.147
HF	9.30%	10.93%	0.641	5.39%	9.52%	0.161
AF	6.98%	6.56%	0.884	4.19%	4.08%	0.961
DM	22.48%	19.13%	0.470	29.34%	19.73%	0.049
LABA therapy	24.21%	29.11%	0.322	1.48%	21.37%	0.004
LAMA + LABA therapy	5.43%	10.93%	0.089	5.39%	6.80%	0.600
ICS + LABA therapy	34.88%	24.04%	0.036	22.16%	49.66%	<0.001
ICS + LABA + LAMA therapy	10.08%	29.51%	<0.001	15.57%	23.13%	0.089

COPD: chronic obstructive pulmonary disease; TB: tuberculosis; FEV_1_: forced expiratory volume in 1 second; FVC: forced vital capacity; FEV_1_/FVC ratio: forced expiratory volume in 1 second/forced vital capacity ratio; BMI: body mass index; HBP: high blood pressure; HF: heart failure; AF: atrial fibrillation; DM: diabetes mellitus; ICSs: inhaled corticosteroids; LABA: long-acting Beta2-agonist; LAMA: long-acting muscarinic antagonist. Data are presented as mean ± standard deviation or percentages. *p*-values indicate statistical significance calculated using Student’s *t*-test for continuous variables or Fisher’s Chi-square test for categorical variables.

**Table 2:** Demographic and clinical characteristics of study participants, comparing controls (No COPD) and COPD cases.

Variable	No COPD (Controls)	COPD (Cases)	Difference (%)/OR	*p* Value
Male gender	59.64%	60.05%	1.02	0.920
Age (years)	48.16 ± 17.86	59.72 ± 11.68	+19.36%	<0.001
TB	31.84%	64.27%	3.85	<0.001
Smoking	53.36%	48.39%	0.82	0.233
Smoking time (years)	29.18 ± 17.10	40.38 ± 11.55	+27.74%	<0.001
Age at 1st smoke (years)	19.33 ± 2.58	24.23 ± 8.65	+20.23%	<0.001
Packs/year	16.39 ± 18.39	15.57 ± 18.33	−5.27%	0.592
BMI	27.32 ± 4.30	27.91 ± 4.32	+2.11%	0.101
Obesity	67.71%	69.23%	1.07	0.695
HBP	8.97%	13.90%	1.64	0.071
HF	6.28%	10.17%	1.69	0.099
AF	4.93%	5.71%	1.17	0.682
DM	24.66%	21.59%	0.84	0.379

COPD: chronic obstructive pulmonary disease; TB: tuberculosis; BMI: body mass index; HBP: high blood pressure; HF: heart failure; AF: atrial fibrillation; DM: diabetes mellitus. Data are presented as mean ± standard deviation or percentages. *p*-values indicate statistical significance calculated using Student’s *t*-test for continuous variables or Fisher’s Chi-square test for categorical variables.

**Table 3:** Propensity score-matched analysis of the association between prior TB and COPD risk stratified by smoking status. Odds ratios and CIs are from weighted logistic regression adjusting for the propensity score, clustering on matched pairs.

	No Smoking (*n* = 312)				Smoking (*n* = 314)			
	O.R.	*p* Value	95% Confidence Interval	ATT	O.R.	*p* Value	95% Confidence Interval	ATT
Prior TB history	2.233	0.001	1.371–3.640	0.18 ± 0.06	5.727	<0.001	3.301–9.937	0.36 ± 0.05
Constant	1.259	0.210	0.878–1.804	-	0.941	0.715	0.681–1.302	-

ATT: Average treatment effect for treated (exposed to TB); i.e., absolute difference in COPD frequency between TB and matched non-TB patients; TB: Tuberculosis.

**Table 4:** Radiological characteristics of pulmonary tuberculosis and associated COPD prevalence and time to first severe exacerbation.

Variable	COPD Prevalence (%)	Time to 1st Severe COPD Exacerbation (Years)
Unilateral TB, no smoking *n* = 58	70.69%	4.97 ± 3.48
Bilateral TB, no smoking *n* = 125	75.20%	4.98 ± 4.18
OR for bilateral TB vs. unilateral TB *p* value	1.26 0.519	−0.003 0.985
Unilateral TB, smoking *n* = 87	39.08%	5.91 ± 4.03
Bilateral TB, smoking *n* = 103	87.38%	6.51 ± 4.94
OR for bilateral TB vs. unilateral TB *p* value	10.79 <0.001	−0.13 0.529
Non-cavitary TB, no smoking *n* = 146	73.97%	5.19 ± 3.90
Cavitary TB, no smoking *n* = 37	72.98%	4.15 ± 4.22
OR for cavitary TB vs. unilateral TB *p* value	0.95 0.902	−0.26 0.222
Non-cavitary TB, smoking *n* = 140	56.43%	5.91 ± 3.92
Cavitary TB, smoking *n* = 50	90.00%	7.11 ± 5.80
OR for cavitary TB vs. unilateral TB *p* value	6.95 <0.001	−0.26 0.173

COPD: Chronic obstructive pulmonary disease; TB: tuberculosis.

## Data Availability

The data presented in this study are available upon request from the corresponding author. The data are not publicly available due to the patient’s personal data protection policy of the University and Hospital.
